# Practical Departments

**Published:** 1905-07-08

**Authors:** 


### PRACTICAL DEPARTMENTS.

### MESSRS. G. N. HADEN AND SONS' HEATING APPARATUS.

Messrs. G. N. Haden and Sons have given a great deal of attention to the problems involved in heating hospital wards, and similar places, for a great many years, and they have acquired a considerable amount of practical experience on the subject of those numerous little nothings that all practical men know are so important. They have made a speciality, among other things, of radiators, for use with all possible agents, hot water, steam, and gas, the latter being a very recent invention. One of their special forms of radiator is known as "The Sanitary," from the fact that it can be thoroughly cleaned in a few minutes. Another speciality is the hinged radiator, which is arranged to be turned out from the wall without moving the whole apparatus. For warming and ventilating wards, another form of radiator, also a speciality, is employed, which may however be made on the hinged pattern if desired. This form of radiator is intended to stand in front of an air inlet, but not to allow the air to pass through between its pipes, as in the sanitary, and other forms. The front of the radiator, that which faces the room to be warmed, is corrugated in section, but there are no openings. The back is formed by a plate of iron on which are cast innumerable small cones. The air from the inlet passes up between these cones, being effectually broken up in the process, and the cones add to the heating surface very considerably. The air passes vertically up on the inner side of the radiator, and is diffused in the room, escaping by outlets provided for it. For heating wards or the rooms of buildings, the supply of water or steam to the radiator is controlled by a very ingenious thermostatic valve, worked by compressed air and ether. The expansion or contraction of the ether sets a supply of compressed air in motion, which actuates the supply-valve to the radiator, the regulation being, it is stated, very perfect, within the limits of the instrument. Messrs. Haden also make numerous forms of heating apparatus and have several special methods of heating water by the latent heat of steam. The firm also make cooking apparatus of all kinds, boiling, steaming, etc. The radiator with the small cones is arranged so that it can be easily cleaned, several parts being movable to allow of this.

### AN APPARATUS FOR SEWER VENTILATION.

Sewer ventilation belongs properly to the department of the Borough Engineer, but there are cases where it is very convenient to be able to apply a system within the area of the hospital boundaries, so as to prevent sewer gases from working back into the hospital wards. Mr. William Farrer of The Star Works, Cambridge Street, Birmingham, has worked out a system, which he has called "The Omnifex," that he claims is applicable to the ventilation of the sewers connected at a single manhole, or to a system of sewers. His idea is to force air into the sewers by the action of the wind, and to cause the air which is passing in, to dilute the sewer gases that are present, and to assist in forcing them out at a height where they will be delivered harmlessly. For this purpose, Mr. Farrer provides a shaft to be fixed at any manhole, the shaft being made double, and having the double office mentioned, of providing fresh air, and of exhausting the sewer gases. Mr. Farrer has worked out a special valve in connection with this system for fixing on the open ends of the inlet drains, connected to manholes and ventilated in a manner which, he claims, while allowing the sewage to pass, prevents the egress of the sewage gases. The double shaft with which the system is provided may be fixed at the junction of more than one drain, branches from the outer tube being taken into each branch of the sewer. The maker claims for his system simplicity and other advantages.

